# Squamous Cell Carcinoma (Marjolin's Ulcer) Arising in a Sacral Decubitus Ulcer Resulting in Humoral Hypercalcemia of Malignancy

**DOI:** 10.1155/2014/715809

**Published:** 2014-08-13

**Authors:** John T. O'Malley, Candace Schoppe, Sameera Husain, Marc E. Grossman

**Affiliations:** ^1^Department of Dermatology, Dermatology Consultation Service, College of Physicians and Surgeons, Columbia University, New York, NY 10032, USA; ^2^City Medical Examiner, New York City Office of Chief Medical Examiner, New York, NY 10016, USA; ^3^Section of Dermatopathology, Department of Dermatology, College of Physicians and Surgeons, Columbia University, New York, NY 10032, USA

## Abstract

Long-standing burns, fissures, and ulcers that undergo malignant transformation into a variety of malignancies, including squamous cell carcinoma, is commonly referred to as a Marjolin's ulcer. It is well recognized that squamous cell carcinomas of the lung and esophagus can cause humoral hypercalcemia of malignancy secondary to paraneoplastic secretion of parathyroid hormone-related peptide. However, it is extremely rare for a squamous cell carcinoma developing in a sacral decubitus ulcer to cause humoral hypercalcemia of malignancy. We describe the first case of a patient found to have elevated serum levels of parathyroid hormone related peptide related to his Marjolin's ulcer. A 45-year-old African American man with T6 paraplegia and a sacral decubitus ulcer present for 20 years was admitted for hypercalcemia of unclear etiology. He was subsequently found to have elevated parathyroid hormone related peptide and an excisional biopsy from the ulcer showed invasive squamous cell carcinoma suggestive of humoral hypercalcemia of malignancy. The patient ultimately succumbed to sepsis while receiving chemotherapy for his metastatic squamous cell carcinoma. Humoral hypercalcemia of malignancy is a rare and likely underrecognized complication that can occur in a Marjolin's ulcer.

## 1. Background

A Marjolin's ulcer is defined as malignant transformation occurring in long-standing burns, fissures, and ulcers. Humoral hypercalcemia of malignancy (HHM) is an important paraneoplastic syndrome occurring in humans with a wide variety of cancers. Parathyroid hormone-related protein (PTHrP) was originally isolated from specific tumors as the primary cause of HHM and is overexpressed by many types of neoplasms. While it has been well recognized that SCC of the lung and esophagus can secrete PTHrP causing HHM, it is rare for a cutaneous SCC to result in HHM with only six reported cases in the literature. We report the first case of a patient with a long-standing sacral decubitus ulcer found to have humoral hypercalcemia of malignancy secondary to the ulcer's transformation to an SCC. This report underscores the importance of maintaining a high level of clinical suspicion for the possibility of a Marjolin's ulcer in a patient who presents with elevated PTHrP and no other evidence of malignancy.

## 2. Materials and Methods

### 2.1. Laboratory Measurements

All laboratory serum specimens were evaluated either at New York Presbyterian-Columbia Hospital (calcium and PTH) (New York, NY) or ARUP laboratories (PTHrP) (Salt Lake City, UT).

### 2.2. Histology

The skin specimen was routinely processed after fixation with formaldehyde and stained with hematoxylin and eosin at the Dermatopathology Laboratory at New York Presbyterian Hospital-Columbia.

## 3. Results and Discussion

**Figure 1:**
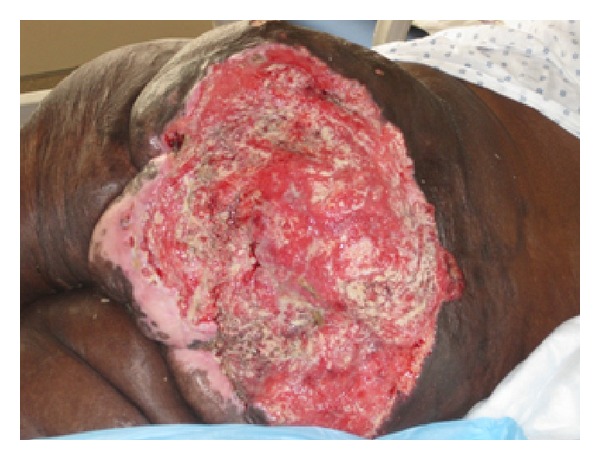
16 × 27.5 cm friable ulcer with fibrinopurulent exudate and exophytic, granulation tissue centrally and peripherally.

A 45-year-old African-American man with T6 paraplegia was admitted for malaise, weight loss, and a sacral decubitus ulcer for 20 years duration. The sacral decubitus ulcer had failed topical wound care and 6 split thickness skin grafts. On admission, he had hypercalcemia (15.4 mg/dL, normal range 8.7–10.2) with low vitamin D 25-OH (18 ng/mL, normal range 30–80), undetectable parathyroid hormone (PTH), and elevated parathyroid hormone related peptide (PTHrP) to 40 pg/mL (normal range 14–27). Cutaneous examination demonstrated a 16 × 27.5 cm friable ulcer with fibrinopurulent exudate covering the sacrum and buttocks with exposure of muscle and bone (Figure 1).

**Figure 2:**
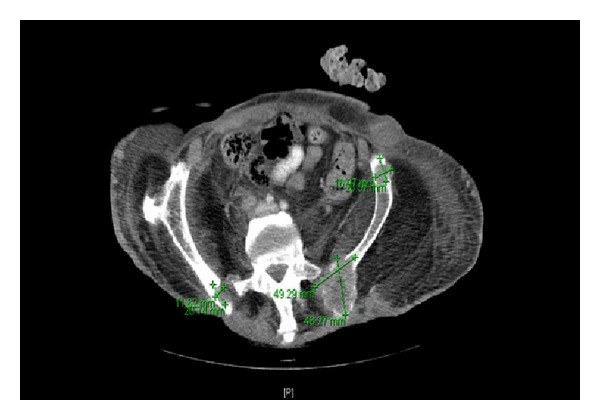
Computed tomography (CT) scan of the abdomen and pelvis demonstrated soft tissue masses with bony destruction of the iliac spines (green lines demarcate bony metastases).

**Figure 3:**
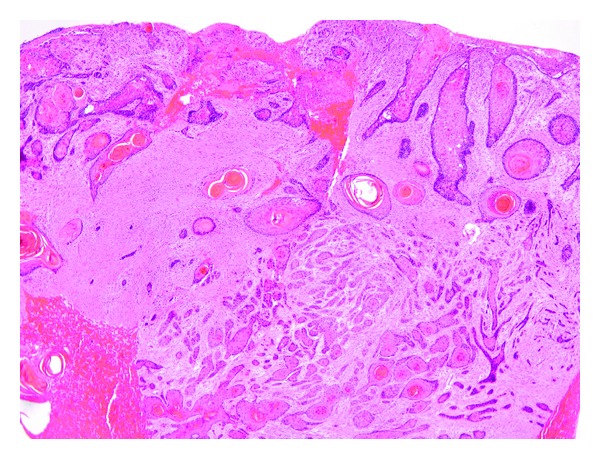
Histopathologic examination with hematoxylin-eosin staining showed epidermal erosion and irregular nests and cords of dysplastic keratinocytes infiltrating the dermis.

A computed tomography scan of the abdomen and pelvis demonstrated soft tissue masses with bony destruction of the iliac spines and the L4 vertebral body (Figure 2). An excisional biopsy of the sacral ulcer was performed which showed epidermal erosion and irregular nests and cords of dysplastic keratinocytes infiltrating the dermis (Figure 3). Fine needle aspiration of the inguinal node showed malignant cells. The neoplastic cells were positive for pancytokeratin, CK5/6, and p63. They were negative for CK7, CK20, TTF-1, CDX-2, PSA, and HPV. The morphology and immunostaining patterns were compatible with squamous cell carcinoma.

The patient refused surgical management and radiation therapy. He was started on cetuximab. Following the third dose, he developed sepsis, was readmitted, and ultimately succumbed to septic shock 1.5 months after the initial dermatology consult. Autopsy showed extensive involvement of the superficial and deep tissues of the lower back, sacrum, buttocks, lower abdomen, groin, upper thighs, and perineum by a high grade SCC with direct extension into the abdominal cavity and marked destruction of the bony pelvis and femoral heads. Lymph node and pulmonary metastases were also identified.

## 4. Discussion

Marjolin's ulcer, first described by the late French surgeon Jean-Nicolas Marjolin in 1828, is the malignant transformation of long-standing burns, fissures, and ulcers, including leprous neurotrophic ulcers, and other chronically inflamed tissue processes [1]. The most commonly associated malignancy is squamous cell carcinoma (SCC) but other neoplasms including basal cell carcinoma and sarcomas have been described. The time necessary for malignant transformation of a sacral decubitus ulcer ranged from 20 to 70 years [2].

Abnormalities of calcium homeostasis are an uncommon complication of Marjolin's ulcer. The amino terminus of parathyroid hormone related peptide (PTHrP) has significant homology to human parathyroid hormone (PTH) which allows it to bind to PTH/PTHrP kidney and bone receptors resulting in humoral hypercalcemia of malignancy. Hypercalcemia occurs because tumor-produced PTHrP interacts with the renal and bone PTH/PTHrP receptor. In the kidney, PTHrP stimulates phosphate excretion and reabsorption of calcium in the distal collecting ducts. In the skeleton, PTHrP induces osteoclastic bone resorption, resulting in elevation of the serum calcium concentration. All normal squamous cells can secrete PTHrP but only certain squamous cell tumors result in hypercalcemia [3]. It is surprising that despite the high incidence of cutaneous squamous cell carcinoma, hypercalcemia is an extremely rare complication of this neoplasm. Indeed, hypercalcemia associated with cutaneous SCC has been reported in fifteen cases [4–6]. Of those fifteen patients, six were found to have increased PTHrP levels strongly supporting the diagnosis of humoral hypercalcemia of malignancy [7]. However, there have been only three published case reports of Marjolin's ulcer associated with hypercalcemia. In one Marjolin's ulcer case, serum levels of PTHrP were normal [5, 8, 9]. We describe the first case of humoral hypercalcemia of malignancy secondary to a Marjolin's ulcer. While the bony destruction from the SCC in our case may have contributed to the patient's hypercalcemia, the finding of concomitantly elevated PTHrP strongly supports the paraneoplastic production of PTHrP as the primary cause of his hypercalcemia. Based on these findings, we recommend surveillance biopsies of long-standing sacral decubitus ulcers in patients found to have hypercalcemia and elevated PTHrP with no known malignancy.

## 5. Conclusions

HHM is a rare and likely underrecognized complication that can occur in a Marjolin's ulcer. Based on this case report and past literature, we recommend surveillance biopsies of long-standing decubitus ulcers to look for malignant transformation to SCC in patients who are found to be hypercalcemic with elevated PTHrP and no other evidence of malignancy.
